# Delivery Methods in Twin Gestations: Evaluating Outcomes, Risk Factors, and the Paradigm Shift Towards Elective Cesarean Deliveries

**DOI:** 10.7759/cureus.46514

**Published:** 2023-10-05

**Authors:** Merin Abraham, Noor Ali, S S Lakshmi Shivani Garapati, Priyanka Pandey, Shreya Nair, Sindhu Swarna, Daddala Vineesha Chowdary, Funmi Aladeniyi, Ananya Daing, Kiran Abbas

**Affiliations:** 1 Internal Medicine, Kasturba Medical College, Manipal, Manipal, IND; 2 Obstetrics and Gynecology, Thumbay University Hospital, Ajman, ARE; 3 General Practice, Dubai Medical College, Dubai, ARE; 4 Obstetrics and Gynecology, Andhra Medical College, Visakhapatnam, IND; 5 Anatomical Sciences, Hind Institute of Medical Sciences, Sitapur, IND; 6 Medicine, Krishna Institute of Medical Sciences, Karad, IND; 7 Medicine, Kasturba Medical College, Mangalore, Mangalore, IND; 8 Medicine, Kamineni Institute of Medical Sciences, Narketpally, IND; 9 Medicine, American University of Antigua, Osbourn, ATG; 10 Medicine, Subharti Hospital, Dehradun, IND; 11 Community Health Sciences, Aga Khan University, Karachi, PAK

**Keywords:** c-section, elective cesarean section, twin gestation, vaginal delivery, fetal loss, intrauterine fetal death, maternal morbidity. maternal death, spontaneous vaginal delivery, cesarean deliveries, twin pregnancies

## Abstract

During the past decade, there has been a consistent rise in the number of twin births and the number of overall cesarean sections (CS) worldwide. This is owed to a variety of social, economic, educational, and scientific factors. More women are opting to advance their professional careers and gain financial stability before having children. Although this approach is understandable, a new set of challenges are faced as a result, the most important of which has been infertility due to advanced maternal age and the subsequent use of assisted fertility treatments, which have been noted to cause multiple gestations. Twin gestations are considered high-risk pregnancies and are associated with an amplitude of potential complications. Arguably, the biggest decision an obstetrician must make when dealing with this population is choosing the most appropriate mode of delivery. Given the lack of clear guidance pertaining to twin deliveries, CSs may often be perceived as safer and are often preferred over vaginal deliveries (VD).

In this narrative review, we aimed to compare the outcomes of different delivery methods (CS versus VD) to investigate whether CS is truly superior to VD. Data were collected from the past two decades and analyzed based on the neonatal and maternal outcomes for each delivery mode. Our results indicate that planned VD is just as safe as CS, if not superior, in most uncomplicated twin pregnancies. Thus, it is best to advise and encourage healthy expecting twin carriers to undergo VD and explore any hesitations or fears they might have. Furthermore, a detailed guideline regarding twin delivery is essential to establish and better navigate twin deliveries, lower the rate of unnecessary CSs, and reduce overall twin gestation morbidity and mortality.

## Introduction and background

The incidence of twin births has experienced a significant upsurge on a global scale. This phenomenon is hypothesized to be interconnected with the escalating employment of reproductive techniques in response to infertility, which affects many individuals [1]. In the United States, twin pregnancies comprise 3.4% of all live births, and among these, 75% are delivered by cesarean delivery [2]. It is anticipated that by 2030, cesarean sections (CSs) will encompass approximately 29% of all childbirths [3]. Despite this increase in CSs, there has been no progress observed regarding maternal and neonatal morbidity and mortality rates [4,5]. A plausible contributor to this escalation in cesarean deliveries could be the elective choice of healthy patients for cesarean procedures, a phenomenon more prevalent in affluent societies [4].

Despite the lack of data demonstrating that CSs are superior to vaginal deliveries (VDs), medical professionals appear to be more lenient in conducting them [6,7]. Up to 10% of deliveries by CS have been linked by the World Health Organization (WHO) to a reduction in maternal and neonatal mortality; rates higher than that have not demonstrated any benefits [8].

Based on the type of conception, twin pregnancies are divided into monozygotic and dizygotic twins. Dizygotic twins are produced by two independent fertilizations, whereas monozygotic twins are produced by a single fertilization that splits into two embryos. Monozygotic twins are further subclassified into monochorionic (MC) or dichorionic (DC) based on the number of placentas and monoamniotic (MA) or diamniotic (DA) based on the number of amniotic sacs. This sub-classification is determined by the time of the zygote's division [1,9].

When compared to singleton pregnancies, twin pregnancies are categorized as high-risk since there is a greater possibility of unfavorable consequences. This is due to the fact that premature births occur in more than half of all pregnancies involving twins, which raises the risk of maternal mortality (MM) by a factor of 2.5 in comparison to pregnancies with only one baby. Carrying two babies instead of one places greater pressure on the mother's body, which can cause physiological modifications that can result in issues for the mother as well as the fetus during the peripartum, intrapartum, and postpartum periods. These complications can be dangerous for both the mother and the baby. Some common issues include maternal hyperemesis gravidarum, which may lead to dehydration and ketosis requiring hospitalization; excessive weight gain; gestational diabetes mellitus; pre-eclampsia/eclampsia leading to HELLP (hemolysis, elevated liver enzymes, and low platelets) syndrome; intrauterine fetal demise (IUFD); placental abruption; premature rupture of membranes (PROM); and preterm labor. Additionally, neonatal respiratory distress syndrome and intracranial hemorrhage are potential complications that could arise during the neonatal phase. Another possible complication is necrotizing enterocolitis. Cerebral palsy is also one of the risks associated with twin pregnancy [10-14].

In pregnancies that involve twins, whether MC or DC, the probability of stillbirth increases compared to pregnancies with a single fetus. Specifically, the likelihood of stillbirth is five times greater for MC twins and 13 times greater for DC twins [15]. Careful monitoring of twin pregnancies is necessary by medical professionals during the antenatal phase. This is especially true for complex twin pregnancies, including monochorionic diamniotic (MCDA) and monochorionic monoamniotic (MCMA) infants, as these situations can be very difficult to handle. Further complicating factors include cord entanglement, twin-twin transfusion syndrome (TTTS), twin anemia polycythemia sequence (TAPS), twin reversed arterial perfusion (TRAP), and selective fetal growth restriction (sFGR) [12,16,17]. The available evidence indicates that obtaining medical assistance from specialized facilities that cater to twins typically results in superior results for both the twin patients and their mother [18].

## Review

Guidelines for management

Most obstetrics and gynecology (OB-GYN) associations have only issued guidelines that ultimately instruct physicians to consider the various factors affecting twin pregnancy. These include but are not limited to parity, gestational age, chronicity, and amnionicity. Thereby, the decision to choose between VD and CS is left up to the physician’s discretion. This has caused many controversies in terms of the best method for delivering twins, particularly the second non-vertex twin. It has led hospitals like Brigham and Women's Hospital (BWH) and King Edward Memorial Hospital, as well as medical associations like The Association of the Scientific Medical Societies (Arbeitsgemeinschaft der Wissenschaftlichen Medizinischen Fachgesellschaften, AWMF) of Germany to compile knowledge and data from multiple sources and come up with their own guidelines [19, 20, 21]. A very recent article published in the American Journal of Obstetrics and Gynecology (AJOG) has seconded this lack as well [22].

**Figure 1:**
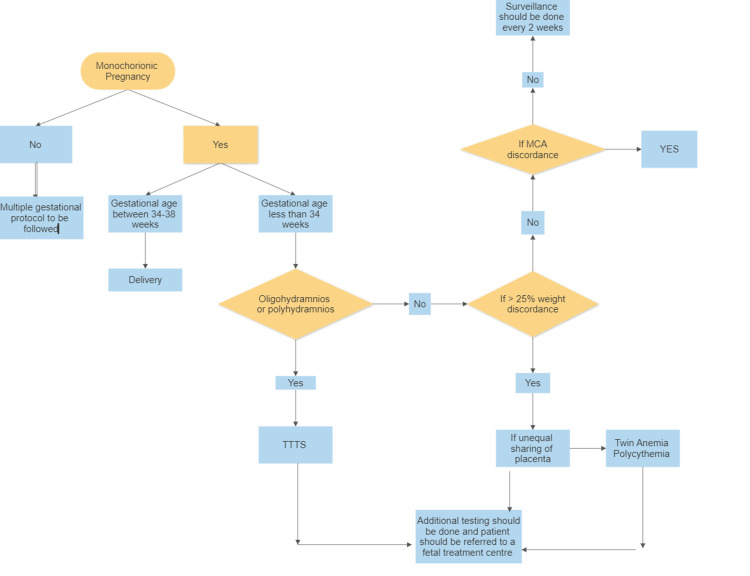
Protocol for the management of twin pregnancies This image has been created by the authors based on the information provided by Jha P. et al. [1]. MCA: middle cerebral artery; TTTS: twin-twin transfusion syndrome

The American College of Obstetricians and Gynecologists (ACOG), the Royal College of Obstetricians and Gynecologists (RCOG), and the International Society of Ultrasound in Obstetrics and Gynecology recommend carrying out an early assessment to ascertain chronicity, amnionicity, and gestational age (GA). Chorionicity warrants meticulous evaluation in cases involving twins. It is noteworthy that these guidelines have been endorsed by both the American College of Radiology and the Society for Maternal-Fetal Medicine, both of which are affiliated with obstetrics and gynecology. Medical experts tally the number of placental masses present and evaluate the thickness of the amniotic sac where it attaches to determine chronicity. This evaluation differentiates between MC and DC pregnancies. The presence of thin membranes, depicted as the "T-sign," indicates that there is only one chorion in the pregnancy. In contrast, thick membranes presented as the "lambda sign" suggest that two chorions are present in the pregnancy. These diagnostic findings are critical in the decision-making process for further management and appropriate prenatal care and to ensure better outcomes for both the mother and the infant [23, 24] (Figure 1).

Outcomes of planned vaginal twin deliveries

Obstetricians encounter a challenging task when managing twin deliveries owing to the fact that twin trials of labor (TOL) tend to result in more adverse birth outcomes compared to singleton deliveries, as indicated by previous studies [25]. There is a variety of literature on the appropriate delivery method for twin pregnancies [26], with guidelines varying across countries and even among different hospitals within the same country. This section will evaluate the existing literature on the maternal and neonatal ramifications associated with vaginal deliveries of twins.

A recent retrospective study was conducted in Germany and involved 274 twin pregnancies to evaluate different modes of delivery and neonatal outcomes. The research encompassed twins born between 32 and 39.4 weeks of gestation from 2015 to 2017. The results indicated that when twins were in vertex presentation with higher birth weights, VD had better success rates compared to other options. However, CS delivery led to more adverse neonatal outcomes for both twins than spontaneous birth did. Specifically, for MCDA pregnancies, the leading twin was more likely to require intubation following cesarean delivery and showed significantly lower AGPAR (appearance, pulse, grimace, activity, and respiration) scores. These findings shed light on prenatal care and labor management strategies that clinicians can utilize to optimize neonatal outcomes in twin pregnancies [26].

In Israel, a retrospective cohort study was carried out involving women who were in the 32nd week of pregnancy or beyond and had twin A in cephalic presentation and no contraindications for vaginal delivery. The comparison group consisted of women with twin pregnancies scheduled for planned cesarean delivery (PCD). The primary objective of this research was to compare maternal and neonatal morbidity between PCD and TOL. Out of 411 twins, 215 underwent TOL, while the remaining 196 underwent PCD. Among the TOL group, the majority (91%, or 196/215) delivered vaginally. Although both groups had similar transfusion rates, postpartum hemorrhage was found to be higher in the TOL group (p < .05). However, there were no significant differences observed in neonatal outcomes between both groups. These findings support the current practice by suggesting that TOL is safe for term twins with the first twin in cephalic presentation without any other contraindications [27].

A detailed investigation was performed at the Feinberg School of Medicine, which is a part of Northwestern University situated in Chicago, Illinois. This study took place in 2008 and utilized a national birth certificate dataset to thoroughly analyze the outcomes of twin gestational deliveries that occurred when the fetus had reached or surpassed 30 weeks gestation. The study specifically focused on vertex/vertex presentations as well as vertex/non-vertex presentations. After an extensive analysis, it was concluded that both vaginal and cesarean deliveries yielded nearly identical rates of complications and fatalities associated with childbirth. Therefore, based on this research, we can accurately state that there is no significant difference between these two methods of delivery in terms of health risks for mothers or babies during twin births [28]. A cohort study was conducted recently on primiparous women who gave birth to their first twin in the vertex position at an urban hospital in Boston between 2007 and 2011. The selection criteria excluded expectant mothers who were below 32 weeks gestation and had medical conditions that made them unsuitable for labor. Depending on the presentation of their second twin, either in the vertex or non-vertex position, delivery and admission assessments of the participants were carried out. It is important to note that those whose second twins were not present in the vertex had comparable or higher rates of vaginal birth when compared to those whose second twins presented vertex-wise. Moreover, approximately 11% of non-vertex twins experienced a change in presentation during childbirth, which contributed to their increased rate of successful VD [29]. In Paris, another study conducted in 2018 concluded that both cephalic and non-cephalic second twin presentations yielded similar outcomes for mothers and newborns during vaginal birth [28].

According to a recent research study [27], it has been suggested that the likelihood of an unsuccessful TOL may be correlated to both the induction process and the gestational age at which delivery occurs. Specifically, there appears to be a higher probability of complications arising during TOL when labor is chemically induced rather than occurring naturally. Further, the risk of an unsuccessful TOL increases as gestational age approaches full term. These findings could have important implications for medical professionals responsible for managing labor and delivery to minimize potential risks and ensure positive outcomes for both mothers and infants. Additionally, independent risk factors for VD in twins were identified as nulliparity and non-cephalic presentation of the second twin, based on research performed at Tampere University Hospital in Finland [25]. It has been confirmed that a safe VD can be implemented in twin pregnancies if certain well-defined conditions are met and appropriate infrastructure and clinical expertise are accessible [26].

Operative vaginal delivery (OVD) in twin pregnancies

Operative vaginal delivery (OVD) involves using medical instruments like forceps and a ventouse (vacuum) to help with safe childbirth in cases of obstructed vaginal labor [30]. An OVD is implicated in instances of a prolonged second stage of labor, inadequate maternal efforts from maternal exhaustion or neuraxial anesthesia, and fetal distress or compromise indicated by a non-reassuring fetal heart tracing [31]. Efficient and expert use of these instruments is required for a successful birth [31].

An OVD is associated with various potential complications. Mothers may suffer perineal injuries, postpartum hemorrhage (which can be traumatic, atonic, or both), shock, disseminated intravascular coagulation (DIC), the need for blood transfusion, femoral nerve injury, sepsis, urinary incontinence, or anal sphincter dysfunction [31]. Infant complications range from asphyxia and facial injuries to cephalohematoma, skull fractures, intracranial hemorrhage, retinal hemorrhage, and Erb's palsy [31].

Research suggests that mothers who undergo OVD have higher rates of postpartum hemorrhage, perinatal birth trauma, sepsis, blood transfusion, and neonatal injuries compared to those who undergo spontaneous vaginal delivery (SVD) [32]. Forceps are often used and include direct forceps and rotational forceps. The use of direct forceps helps overcome problems of cup detachment with a vacuum and was associated with lower rates of cephalohematoma and retinal hemorrhages, especially in premature fetuses [33]. Rotational forceps allow for rotation of the fetus’s head, yielding the greatest number of successful OVDs, followed by manual rotation and direct forceps, respectively [34]. In contrast, with vacuum use, the chances of failed rotation and an unsuccessful OVD were high [35].

Nonetheless, forceps caused greater maternal trauma, namely cervical and vaginal lacerations, urethral and anal sphincter injuries with subsequent incontinence, and hematomas, and thus were associated with higher rates of postpartum hemorrhage, sepsis, and DIC when compared to vacuum-assisted deliveries [33]. It’s worth noting that birth weight did not appear to affect these vacuum delivery complications, and there have been no recordings of severe long-term neurological disabilities from the complications encountered in vacuum deliveries with pre-term babies [36,37].

The BD Odon Device is a recent invention (an inflatable circular air cuff attached to a thin circumferential polyethylene sleeve) that promises improvements in maternal and neonatal OVD outcomes when compared to forceps and vacuums [38]. However, this device is still undergoing trials and is not yet on the market for clinicians to use [38].

Outcomes of VD based on the inter-twin delivery interval 

Studies reveal that inter-twin delivery intervals exceeding 10 minutes are associated with an increased likelihood of internal podalic version, OVD, and CS for the second twin [39]. Inter-twin delivery intervals of >10 minutes led to fetal hypoxia and subsequent lower umbilical artery pH <7.15, as well as poorer APGAR scores <7 in the second twin [39]. The fall in umbilical artery pH and AGPGAR scores remained the same between MC and DC twins with prolonged inter-twin delivery intervals [40]. Interestingly, the likelihood of adverse neonatal outcomes and neonatal intensive care unit (NICU) admissions for the second twin increased with a decrease in the umbilical artery pH level of the first twin [40]. In addition, a birth weight difference exceeding 25% was linked to adverse neonatal outcomes in the second twin [41]. A disparity in the two characteristics of the twins was found to be insignificant [40]. Abnormal cardiotocography (CTG) was recorded as a predicting factor for fetal hypoxia when comparing the outcomes of twins with constant inter-twin delivery intervals [42].

Outcomes of CS in twin delivery

For many obstetricians, performing a CS in twin pregnancies is less complicated than a VD, especially given the lack of clear guidelines from OB-GYN associations, namely ACOG and RCOG, specifically in cases of non-cephalic presenting twins [43]. Many factors are considered when deciding the mode of delivery, one of which is parity. In general, a nulliparous woman has a less successful chance of delivering twins vaginally than a multiparous woman [44]. Furthermore, a couple of large older cohort studies of twins found lower adverse perinatal risk with elective CSs when compared to emergency CS or VD [45-47]. As such, reviewing studies looking at C-section outcomes in twin pregnancies would be the next best step in evaluating the true efficiency of C-sections and compiling satisfactory evidence to support or dispute this practice. When discussing these outcomes, we assess two aspects: maternal outcomes and neonatal outcomes.

In 2013, a controlled and randomized study, "The Twin Birth Trial," was conducted across various centers globally. The study's objective was to examine the outcomes of twin deliveries via planned CS versus planned VD in women between 32 and 38 weeks of pregnancy during their third trimester. The analyzed data indicated no statistically significant increase or decrease in fetal or neonatal mortality rates or critical infant illness when planning for a cesarean section during pregnancy. Notably, the research emphasized the importance of delivering twins vaginally, especially if the presenting twin is the vertex [48]. Over the years that followed, the Twin Birth Trial underwent multiple secondary analyses. One of these took place in 2018 and involved an examination of 1,435 participants who went into spontaneous labor. This analysis found no significant differences between planned VD and planned CS in terms of neonatal or maternal outcomes [49]. Another study was conducted in 2020, which examined the outcomes of 1,347 participants who required delivery prior to the onset of labor through either induction of labor (IOL) or pre-labor C-section (PrlCS). The results were comparable to those obtained from the original study, with one notable exception: PrlCS produced better maternal outcomes due to its associated lower risk of adverse maternal effects [50]. A supplementary secondary analysis was carried out in 2021 to assess the intended method of delivery and its impact on GA at birth. The results of this evaluation revealed that opting for a vaginal birth plan led to fewer negative perinatal consequences within the 32-37-week GA timeframe, while a CS performed after 37 weeks demonstrated greater safety [51]. The previously mentioned information is consistent with the JUmeaux MODe d'Accouchement (JUMODA) research carried out in France during the period between February 2014 and March 2015. This study involved a cohort of 5,915 women and aimed to compare neonatal mortality and morbidity outcomes for twin pregnancies under planned VD and planned CS. It was a prospective population-based cohort study that showed better results during scheduled VDs at or beyond the 32nd week of gestation. However, findings indicate a greater risk for adverse neonatal outcomes in cases of planned CS before week 37. The results have significant implications as they emphasize the importance of identifying the optimal mode and time of delivery for twin pregnancies, which can substantially impact maternal and child health outcomes [52].

Outcomes of combined vaginal-cesarean delivery of the second twin

Multiple studies comparing CS and VD in twin pregnancies concluded that women presenting with premature labor prior to 37 weeks gestation were more likely to undergo a CS for the second twin. In addition, a CS to deliver the second twin occurred more commonly in women who did not require an induction or oxytocin drip to deliver the first twin vaginally. These observations suggest that the method of delivering the first twin significantly influences the method of delivering the second twin [53]. Furthermore, maternal morbidity, postpartum hemorrhage (PPH), and endometritis have been noted more frequently in those undergoing CSs for both twins and for the second twin than in those delivering both twins vaginally [53]. Again, this emphasizes the importance of intrauterine maneuver skills in increasing the rates of successful VD in both twins, lowering the overall CS rate in twins, and improving maternal morbidity in twin pregnancies [53]. To further drive this point home, multiple sources have found no discernible correlation between delivering non-cephalic second twins vaginally and an increased probability of neonatal morbidity [28,54]. In fact, the outcomes following an internal podalic version to deliver the second twin were more favorable than those of a combined delivery [55].

Geographical disparity in outcomes of vaginal versus cesarean twin deliveries

The outcomes of twin pregnancies vary significantly based on differing socio-economic statuses. Countries with lower socioeconomic status have worse maternal and neonatal outcomes. A study examining 60 low- and moderate-income countries (LMICs) found that twins born vaginally at healthcare facilities were more likely to experience early neonatal mortality than twins delivered via CS. This risk was more evident in nations with increased CS rates (>15%). As a result, the authors suggest that in countries with lower socioeconomic status and lower CS rates (<15%), the protective effects of CS might be compromised due to various reasons. Most importantly, there is inadequate emergency access to operation rooms, a lack of skilled obstetricians, an undersupply of surgical equipment, and the absence of appropriate medical infrastructure [56]. Another study examined 21 cases of twins delivered beyond 34 weeks of gestation in LMICs and concluded that PrlCS was associated with reduced chances of neonatal near misses compared to SVD. Additionally, lower rates of neonatal and perinatal mortality were observed with PrlCS when the first twin presented non-vertex. Rates of PrlCS were particularly higher in more affluent LMICs [57].

In Portugal, a retrospective cohort study analyzed the outcomes of 207 MCDA twins and observed higher rates of NICU admissions and lower APGAR scores in neonates born via CS compared to neonates born vaginally [58]. Similarly, it was found that LMIC cesarean deliveries for twins have higher incidences of severe adverse maternal outcomes [59]. The prevalence of these outcomes varied significantly across different regions. Of note, Africa too had elevated risks associated with CSs in twin pregnancies due to the limited number of secure and well-established hospitals that offer CSs. In contrast, Latin America demonstrated relatively lower risks given the higher utilization of CS procedures. These findings call attention to regional wealth as a factor in overcoming the adversities associated with twin birth [59].

## Conclusions

Twin gestations are commonly accompanied by a myriad of maternal and neonatal complications and are thus considered high-risk pregnancies. As a result, decision-making becomes challenging, especially that pertaining to delivery. Many elements factor into deciding the mode of delivery, such as gestational age, chronicity and amnionicity, fetal position, fetal weight, and overall maternal and fetal health. Although patient preferences are honored and shared decision-making is encouraged, physicians must fulfill their ethical duty of doing no harm. In this case, that means avoiding all unnecessary CSs. Patients should be counseled in detail about the differences between VD and CS, the potential complications, downtime, and recovery for them and the baby. The physician must also do their best to clear up any misconceptions and address any fears the patient might have regarding VD.

Based on the data collected, we suggest considering the following when planning twin deliveries:

1. As of now, in general, most studies do not deem CS superior to VD in terms of maternal and neonatal outcomes; 2: Schedule vaginal births between 32 and 37 weeks of gestation; granted, there are no contraindications to vaginal birth; 3: A non-vertex-presenting second twin should not in itself be a cause for undergoing a CS; 4: Ensure labor and delivery (L&D) personnel are adequately trained in intrauterine maneuvers, such that both twins are delivered vaginally when attempting a vaginal birth, to avoid delivering the second twin via CS; 5: Refrain from using operative vaginal instruments unless necessary and limit the inter-twin delivery interval to <10 minutes; 6: When contraindications to vaginal birth exist, schedule CSs after 37 weeks, unless an emergency delivery arises before that.

It is necessary to establish an in-depth guideline addressing criteria for choosing the most optimal mode of delivery in twin pregnancies. Although obstetricians will still need to use their clinical expertise and evaluate patients on a case-to-case basis, the presence of a precise guideline will immensely help ease the decision-making process and ultimately build physicians' confidence in dealing with twin gestations. Establishing this guideline requires large-scale studies comparing the outcomes of VD and CS in twin pregnancies. These studies should focus on the effects of every factor (parity, gestational age, chronicity, and amnionicity) in isolation on the mode of delivery. Cost, participant recruitment, and obtaining ethical approvals would be the main obstacles to the research initiatives suggested.

Lastly, most studies considered in this review were conducted in first-world countries; hence, this review does not accurately reflect the status of VD versus CS in less-developed countries.
